# Supplementary material for: Predicting multiple conformations via sequence clustering and AlphaFold2
Source: Nature. 2023 Nov 13;625(7996):832–9. doi: 10.1038/s41586-023-06832-9 (PMC10808063; doi:10.1038/s41586-023-06832-9)
Supplement: Supplementary file 1 — Supplementary Discussion, including Supplementary Figs. 1 and 2. [file 41586_2023_6832_MOESM1_ESM.pdf]

nature portfolio

https://doi.org/10.1038/s41586-023-06832-9

Supplementary information

Predicting multiple conformations via sequence clustering and AlphaFold2

In the format provided by the authors and unedited

Supplemental Discussion corresponding to "**Predicting multiple conformational states via sequence clustering and AlphaFold2**"

Investigating intrinsic thermodynamics vs. local evolutionary couplings in AF2 predictions. Two effects could be driving AF2 predictions based on these "shallow" MSA clusters. One effect is that these shallow clusters could be somehow better allowing the primary sequence to influence AF2's output. Another mode is that the shallow clusters themselves could contain local coevolutionary couplings. We investigated this for $KaiB^{RS}$ and $KaiB^{TV}$-4 by predicting their structures with no MSA (Supplemental Figure 1a). Whereas $KaiB^{RS}$ is also predicted in the ground state with no MSA, intriguingly, $KaiB^{TV}$-4 is incorrectly predicted to also be in the ground state using just a single sequence, even though using the closest 10 sequences as an MSA predicts the FS state. Furthermore, none of the 10 sequences in the shallow MSA, when themselves predicted in single-sequence mode, have high-confidence models for the FS state (Supplemental Figure 1b).

We generated 10 samples from the same 10-sequence MSA where we shuffled residues within columns to preserve amino acid content at each position but ablate any coevolutionary information. Of these samples, 1/10 was in the FS state, though 9/10 contained the C-terminal helix characteristic of the FS state. However, all were lower pLDDT than the original model. This indicates that coevolutionary information within the "shallow" MSA for $KaiB^{TV}$-4 is important in directing AF2 to predict the FS state with high confidence from the shallow MSA (Supplemental Figure 1c).

We investigated the extent to which this plays a role across the whole phylogenetic tree of KaiB by making single-sequence predictions for all the variants and comparing the states of these predictions to the states predicted using shallow MSAs. Roughly 50% of sequences predicted in the FS state using shallow MSAs switched to a different structure when using single-sequence mode (Supplemental Figure 1d).

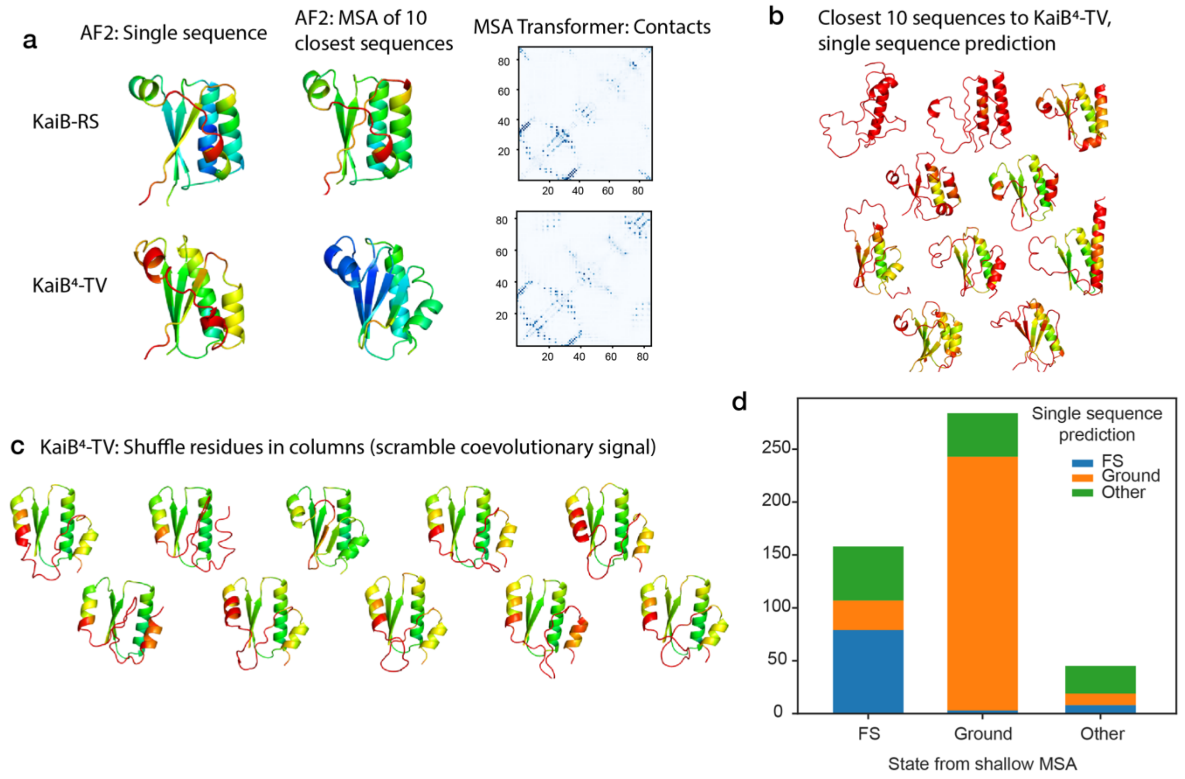

Supplemental Figure 1. a) KaiB^{RS} is predicted in the ground state both in single-sequence mode and using the closest 10 sequences. However, KaiB^{TV}-4 is predicted incorrectly in the ground state in single-sequence mode, but correctly in the FS state using the closest 10 sequences. Right: MSA Transformer contact maps agree with the states predicted by the closest 10 sequences. b) Predictions of each of the 10 closest sequences to KaiB^{TV}-4 in single-sequence mode. None of the sequences have high confidence for the FS state. c) 10 samples of the closest-10-sequences MSA for KaiB^{TV}-4, shuffling residues within columns to preserve amino acid content but destroy coevolutionary information. All models contain the C-terminal helix, but just 1/10 has the C-terminal FS state beta-strands. None have high confidence for the FS state. Coloring in a-c by pLDDT. d) Comparing models of KaiB variants in the phylogenetic tree in Fig. 2a predicted using shallow MSAs and in single-sequence mode. For variants predicted in the FS state using shallow MSAs, roughly 50% are predicted in the FS state structure in single-sequence mode, indicating that coevolutionary signal is important for predicting the FS state even in shallow MSAs.

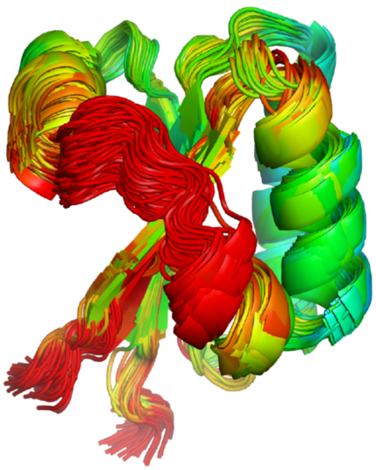

Supplemental Figure 2: Introducing non-sequence-based forms of noise by sampling across models, random seeds, and with dropout does not give any sampling of the FS state for KaiB^{RS}. Coloring by pLDDT.

Table S1. Codon-optimized DNA sequences, protein sequences, and primers for site-directed mutagenesis for KaiB^{RS} and KaiB^{TV}-4 constructs.

Table S2. PDB entry data corresponding to proteins investigated as matches to either original state or alternate state.
