# Supplementary material for: Predicting multiple conformations via sequence clustering and AlphaFold2
Source: Nature. 2023 Nov 13;625(7996):832–9. doi: 10.1038/s41586-023-06832-9 (PMC10808063; doi:10.1038/s41586-023-06832-9)
Supplement: Supplementary file 2 — Reporting Summary [file 41586_2023_6832_MOESM2_ESM.pdf]

nature portfolio

Corresponding author(s): Dorothee Kern

Last updated by author(s): Oct 14, 2023

Reporting Summary

Nature Portfolio wishes to improve the reproducibility of the work that we publish. This form provides structure for consistency and transparency in reporting. For further information on Nature Portfolio policies, see our Editorial Policies and the Editorial Policy Checklist.

Statistics

For all statistical analyses, confirm that the following items are present in the figure legend, table legend, main text, or Methods section.

n/a	Confirmed	
☐	☒	The exact sample size (*n*) for each experimental group/condition, given as a discrete number and unit of measurement
☒	☐	A statement on whether measurements were taken from distinct samples or whether the same sample was measured repeatedly
☐	☒	The statistical test(s) used AND whether they are one- or two-sided<br>*Only common tests should be described solely by name; describe more complex techniques in the Methods section.*
☒	☐	A description of all covariates tested
☒	☐	A description of any assumptions or corrections, such as tests of normality and adjustment for multiple comparisons
☐	☒	A full description of the statistical parameters including central tendency (e.g. means) or other basic estimates (e.g. regression coefficient) AND variation (e.g. standard deviation) or associated estimates of uncertainty (e.g. confidence intervals)
☐	☒	For null hypothesis testing, the test statistic (e.g. *F*, *t*, *r*) with confidence intervals, effect sizes, degrees of freedom and *P* value noted<br>*Give P values as exact values whenever suitable.*
☒	☐	For Bayesian analysis, information on the choice of priors and Markov chain Monte Carlo settings
☒	☐	For hierarchical and complex designs, identification of the appropriate level for tests and full reporting of outcomes
☐	☒	Estimates of effect sizes (e.g. Cohen's *d*, Pearson's *r*), indicating how they were calculated

Our web collection on statistics for biologists contains articles on many of the points above.

Software and code

Policy information about availability of computer code

Data collection	NMR data collection: VnmrJ version 4.2<br>Size Exclusion Chromatography coupled to Multi Angle Light Scattering (SEC-MALS) data collection: Astra 8.1.2.1
Data analysis	NMR analysis and structure determination:<br>NMRPipe: v11.5<br>SMILE: version 11.5<br>POKY: build_02-13-2023j<br>XPLOR: version 3.5<br>PONDEROSA C/S: Build_04-26-2018<br>PINE-SPARKY.2: 2018<br>APES and iPICK: internal to POKY<br>TALOS-N: Version 4.12<br>AUDANA internal to PONDEROSA C/S<br><br>The AF-Cluster code is publicly available at https://github.com/HWaymentSteele/AF_Cluster. Its dependencies are<br>scikit-learn v.1.1.1<br>scipy v1.7.3<br>Biopython v1.81<br>mdtraj v1.9.9<br>numpy v1.21.5<br>pandas v1.4.3

polyleven v0.8

Phylogenetic tree development:
BLASTP: 2.6.0
MAFFT: v.7
RaxML: v8.2.9
PAML: v.3.3.20170116
CDHIT: v4.8.1

For manuscripts utilizing custom algorithms or software that are central to the research but not yet described in published literature, software must be made available to editors and reviewers. We strongly encourage code deposition in a community repository (e.g. GitHub). See the Nature Portfolio guidelines for submitting code & software for further information.

Data

Policy information about availability of data

All manuscripts must include a data availability statement. This statement should provide the following information, where applicable:

- Accession codes, unique identifiers, or web links for publicly available datasets
- A description of any restrictions on data availability
- For clinical datasets or third party data, please ensure that the statement adheres to our policy

Data corresponding to all AF-Cluster modeling and analysis presented here are publicly available at www.github.com/HWaymentSteele/AF_Cluster. The NMR assignments of KaiBRS, KaiBRS-3m, and KaiBTV-4 have been deposited in the Biological Magnetic Resonance Bank (BMRB) under accession codes 52018, 52017, 52019, respectively. The NMR structure of KaiBTV-4 is available at PDB accession code 8UBH and BMRB accession code 31107.

Research involving human participants, their data, or biological material

Policy information about studies with human participants or human data. See also policy information about sex, gender (identity/presentation), and sexual orientation and race, ethnicity and racism.

Reporting on sex and gender	N/A
Reporting on race, ethnicity, or other socially relevant groupings	N/A
Population characteristics	N/A
Recruitment	N/A
Ethics oversight	N/A

Note that full information on the approval of the study protocol must also be provided in the manuscript.

Field-specific reporting

Please select the one below that is the best fit for your research. If you are not sure, read the appropriate sections before making your selection.

☒ Life sciences ☐ Behavioural & social sciences ☐ Ecological, evolutionary & environmental sciences

For a reference copy of the document with all sections, see nature.com/documents/nr-reporting-summary-flat.pdf

Life sciences study design

All studies must disclose on these points even when the disclosure is negative.

Sample size	No statistical methods were used to determine sample size.
Data exclusions	Residues with significant overlap in the HSQC spectra were excluded from population estimates for the KaiBRS and KaiBRS-3m constructs in figure 3e. This data exclusion practice was pre-established.
Replication	Beyond the first KaiB example, the AF-cluster was tested on a diverse sample of 5 other experimentally-validated fold-switching proteins. NMR experiments were performed once.
Randomization	No randomized sample selection was applied.
Blinding	Blinding was not relevant for this study as the work was done with protein samples of known compositions.

Reporting for specific materials, systems and methods

We require information from authors about some types of materials, experimental systems and methods used in many studies. Here, indicate whether each material, system or method listed is relevant to your study. If you are not sure if a list item applies to your research, read the appropriate section before selecting a response.

Materials & experimental systems

n/a	Involved in the study
☒	☐ Antibodies
☒	☐ Eukaryotic cell lines
☒	☐ Palaeontology and archaeology
☒	☐ Animals and other organisms
☒	☐ Clinical data
☒	☐ Dual use research of concern
☒	☐ Plants

Methods

n/a	Involved in the study
☒	☐ ChIP-seq
☒	☐ Flow cytometry
☒	☐ MRI-based neuroimaging
